# Ampleur et impact des évènements indésirables graves liés aux soins: étude d'incidence dans un hôpital du Centre-Est tunisien

**DOI:** 10.11604/pamj.2013.16.68.1161

**Published:** 2013-10-25

**Authors:** Nabiha Bouafia, Iheb Bougmiza, Fathi Bahri, Mondher Letaief, Pascal Astagneau, Mansour Njah

**Affiliations:** 1 CHU Farhat Hached de Sousse-Tunisie; 2 Département de médecine préventive et communautaire, Faculté de médecine de Sousse, Tunisie; 3 Service d'épidémiologie, CHU Fattouma Bourguiba de Monastir, Tunisie; 4 Centre de coordination et de lutte contre les infections nosocomiales (CCLIN) Paris Nord, France

**Keywords:** Evènements indésirables graves, infections associées aux soins, soins invasifs, gestion des risques, sécurité des patients, Serious Adverse Events, healthcare associated infections, invasive care, Risk Management, Patients safety

## Abstract

**Introduction:**

La prévention des événements indésirables représente une priorité de santé du fait de leur fréquence et de leur gravité potentielle. Ce travail a été mené afin d'avoir un diagnostic de la situation épidémiologique relative aux événements indésirables survenant dans notre hôpital.

**Méthodes:**

Une étude prospective a été menée auprès de tous les patients qui ont été hospitalisés au CHU Farhat Hached - Sousse (Tunisie) sur une période d'un mois dans quatorze services de l'hôpital. La détection d'évènement indésirable grave (EIG) était basée sur les critères adoptés dans différentes études. Les tests T et Chi 2 ont été utilisés pour identifier les facteurs contribuant à l'apparition d'évènements indésirables.

**Résultats:**

Au total, 162 EIG ont été identifiés pendant la période. 45% de ces évènements étaient des infections nosocomiales. Ces EIG ont eu comme conséquences un décès chez 9,2% des patients, la mise en jeu du pronostic vital de 26% des patients et la prolongation de la durée de séjour chez 61,7% d'entre eux. L'admission dans des circonstances particulières et l'exposition à des soins invasifs étaient identifiés comme des facteurs de risque potentiels EIG.

**Conclusion:**

Le renforcement de la stratégie de gestion des risques sanitaires en ciblant préférentiellement le risque infectieux constitue une étape fondamentale dans l'amélioration de la sécurité des patients au sein de notre établissement de santé.

## Introduction

La notion d'évènements indésirables date des années 50 mais n'attiraient pas autant l'attention qu'aujourd'hui. Le rapport « to err is human » publié par l'institut de médecine américain vers les années 90 a en effet représenté un catalyseur dans la prise de conscience de l'importance du thème de la sécurité du patient dans les établissements de santé en estimant entre 44000 et 98000 le nombre de décès annuels liés à une erreur médicale évitable [1]. Depuis, la détection et l'analyse systématique des évènements indésirables liés aux soins sont devenues un sujet d'étude important en matière d'amélioration continue de la qualité des soins et de gestion des risques à l'hôpital [2–5]. Ainsi, les données épidémiologiques sur l'ensemble des évènements indésirables graves (EIG) recueillies dans le cadre d'une revue systématique des différentes études menées aux Etats-Unis, en Australie, au Canada, en Grande-Bretagne, en Nouvelle-Zélande et au Danemark, ont montré que le taux d'incidence des patients avec des EIG pris en charge dans les établissements de santé de court séjour variait entre 3 et 17% avec une médiane de 9,2%. Près de 27% à 69,6% de ces EIG étaient considérés comme évitables ou conséquences d'une négligence [6–12].

En France, une étude nationale sur les EIG liés aux soins (ENEIS) réalisée en 2004 a montré une densité d'incidence des EIG survenant au cours de l'hospitalisation de 6,6/1000 jours d'hospitalisation dont 2,3/1000 jours d'hospitalisation auraient pu être évités selon l'expertise médicale [13] Ces événements seraient à l'origine d'un décès dans 5 à 14% des cas et d'une incapacité d'au moins un mois pour 16 à 44% d'entre eux [14]. Selon Kohn L.T, les taux de décès imputables aux EIG seraient ainsi supérieurs à ceux induits par le VIH ou le cancer du sein. Le coût total de la prise en charge des EIG considérés évitables aux Etats-Unis en 1999 a été évalué entre 17 milliards de dollars et 29 milliards de dollars chaque année [11, 15].

Ces évènements entrainent en outre une prolongation de la durée de séjour hospitalier estimée à 1 521 jours d'hospitalisations supplémentaires chez les patients hospitalisés dans un établissement de santé canadien [9]. En Tunisie, nous disposons de très peu de données épidémiologiques sur les différentes formes que peuvent prendre les évènements indésirables liés aux soins. Ainsi, une première étude d'incidence rétrospective des EIG liés aux soins réalisée au CHU de Monastir en 2005 a montré une incidence autour de 10% dont 60% ont été jugés évitables [16]. Grâce à une approche plutôt prospective, notre travail vise à déterminer l'ampleur et la nature des EIG de soins apparus au cours de l'hospitalisation au CHU Farhat Hached de Sousse afin de mieux orienter la stratégie de leur prévention.

## Méthodes

L'étude a eu lieu durant l'année 2009 au CHU Farhat Hached de Sousse, ville du Centre-Est tunisien, comportant 668 lits avec un taux d'occupation de 75,3%. L'établissement de santé, situé au c'ur de la ville, dessert avec un autre CHU de même capacité et 3 cliniques privées une population de 600 000 habitants.

### Définition de l'EIG

Nous avons adopté les définitions standards utilisées dans les différentes études sur les EIG [6–8, 10, 12, 13]. Un événement indésirable est ainsi un évènement clinique ou para clinique, non désiré (défavorable) pour le patient, imputable aux soins (stratégies et actes de traitement, de diagnostic, de prévention et de réhabilitation) et non à l'évolution naturelle de la maladie. Il est considéré comme grave s'il était associé à un décès, et/ou à une menace vitale et/ou à une incapacité à la fin de l'hospitalisation et/ou à une prolongation de la durée d'hospitalisation d'au moins un jour. La prolongation de la durée de séjour a été portée suite à l'avis de l'expert qui a jugé que, le patient aurait pu quitter l'établissement au jour (J - X), si l'évènement indésirable grave ne serait pas survenu. (X = le nombre de jours supplémentaires causés par la survenue de l'EIG, non chiffré dans notre étude).

### Type et population d'étude

Il s'agit d'une étude descriptive, prospective, d'incidence, intéressant tous les patients qui ont été hospitalisés dans quatorze services du CHU Farhat Hached de Sousse (9 services médicaux, 3 services chirurgicaux, 1 service de gynéco-obstétrique et 1 service de pédiatrie) et suivis pendant un mois durant l'année 2009. Ont été exclus les services de néonatologie, de psychiatrie, les hospitalisations de jour dans tous les services et les post partum de moins de 48h suite à des accouchements par voie basse. Par ailleurs, n'ont étaient inclus dans notre étude que les EIG apparus ou identifiés pendant l'hospitalisation et durant la période d'observation.

### Recueil des données

L'étude a été effectuée à l'aide d'un questionnaire pré-testé rempli, par des médecins préalablement formés à la méthodologie de recueil, à chaque passage dans les services, selon un calendrier prédéfini. Six passages ont été effectués dans chaque service durant la période d'étude. Certaines variables ont été collectées à l'admission, en particulier, les caractéristiques générales du patient (âge, sexe, …), son profil clinique à l'admission (antécédent d'hospitalisation dans les six derniers mois, immunodépression, …) et les caractéristiques de l'admission (programmée ou en urgence,). D'autres variables ont été recueillies lors de chaque passage, en se référant au dossier médical et au médecin traitant, en particulier l'exposition aux procédures de soins invasifs diagnostiques ou thérapeutiques (chirurgie, biopsie…) aux dispositifs médicaux (cathéter veineux périphérique, sonde urinaire…) et aux produits de santé (médicaments, produits de réhydratation, produits sanguins et produits diététiques).

Pour la détection d'un EIG nous nous sommes référés à un certain nombre de critères, testés et validés dans la littérature [7, 9, 11, 13], néanmoins adaptés aux objectifs de notre étude. Ces critères au nombre de neuf figurent en annexe A. Un EIG était suspecté lorsqu'au moins un des critères était rempli. A la fin du recueil, tous les EIG suspectés ainsi que leurs conséquences ont été réexaminés et validés par un médecin expert (Professeur en médecine) et extérieur à l'étude.

### Analyse des données

L'analyse statistique a été réalisée en utilisant le logiciel EPI INFO. Nous avons calculé le taux d'incidence d'au moins un EIG par patient en rapportant le nombre de patients ayant présenté au moins un EIG durant leur hospitalisation et pendant la période de recueil sur le total de patients qui étaient hospitalisés dans les services inclus durant la même période. La durée de séjour n'a pas pu être remplie pour tous les patients rendant ainsi difficile le calcul d'une densité d'incidence. Afin d'étudier la part de certains facteurs dans la genèse d'EIG, nous avons utilisé les tests statistiques appropriés (le test de Student pour comparaison des moyennes et le test de Chi2 pour la comparaison des fréquences). Le seuil de significativité (p) était fixé à 0.05.

## Résultats

### Incidence et typologie des EIG

**Figure 1:**
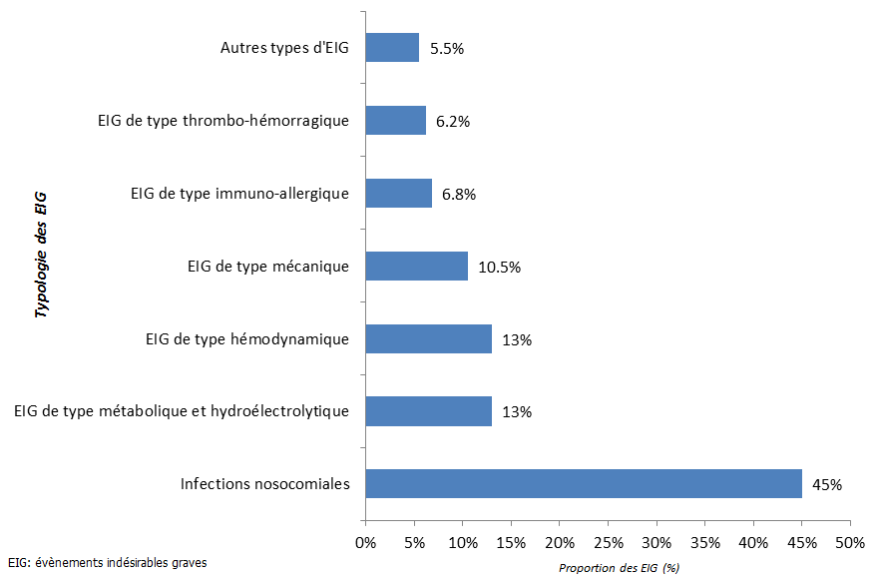
Typologie des évènements indésirables graves selon leur présentation clinique

Durant la période d'un mois d'étude, 162 patients parmi ceux qui étaient hospitalisés et suivis dans les services inclus à l'étude (N = 1 428) ont présenté au moins un EIG confirmé par l'expert médical. Rapporté au total des patients admis pendant la période d'étude, l'incidence d'au moins un EIG par patient est de 11,3% (IC 95% {9.6 - 12.9}). Ces EIG prédominent dans les unités de soins intensifs où un tiers des patients (33.3%) ont développé au moins un EIG durant leur séjour (Tableau 1). En dehors de la pédiatrie et la gynéco-obstétrique, cette incidence est de 13.7% dans les services médicaux et 8.9% dans les services chirurgicaux (Tableau 1). Tous les EIG recensés ont été classés selon leurs présentations cliniques. La Figure 1 montre que ce sont les infections nosocomiales qui occupent le premier rang des EIG dans notre hôpital avec une proportion de 45% suivies par les évènements de type hémodynamiques et métaboliques à des proportions égales (13%), mais certes, beaucoup moins élevées que celles de type infectieux. D'autres catégories d'EIG ont été identifiées dans notre étude telles que les évènements de type mécaniques (10.5%), immuno-allergiques (6.8%) et thrombo-hémorragiques (6.2%) (Figure 1). Des exemples de chacune de ces catégories d'EIG sont présentés en Annexe B.

**Tableau 1:** Incidence des évènements indésirables graves selon la spécialité

Spécialités	Nombre de patients hospitalisés	Nombre d'EIG	Incidence d'EIG (%)
Unités de soins intensives	30	10	33.3
Services médicaux	665	91	13.7
Services chirurgicaux	439	39	8.9
Pédiatrie	81	10	12.3
Gynéco-obstétrique	213	12	5.6
Total	1428	162	11.3

### Conséquences des EIG

Plus de la moitié des EIG (61,7%) ont entrainé une prolongation de la durée de séjour et près d'un quart ont engagé dans l'immédiat le pronostic vital du patient avec une évolution favorable ultérieurement (Tableau 2). Par ailleurs, quinze EIG étaient associés à la survenue d'un décès soit une proportion de 9,2%, sans l'être imputable de façon formelle (Tableau 2). Enfin, l'incapacité physique telle qu'une parésie faciale post parotidectomie ou l'amputation mi-jambe suite à une prise en charge non adaptée d'un abcès malléolaire chez un diabétique, était observée dans 3% des cas (Tableau 2).

**Tableau 2:** Conséquences des évènements indésirables graves

Conséquences des EIG	Nombre d'EIG	Fréquence relative (%)
Prolongation de la durée de séjour	100	61.7
Mise en jeu du pronostic vital	42	26
Incapacité physique	5	3
Décès	15	9.3

### Facteurs contribuant à la survenue d'EIG

L'analyse des caractéristiques démographiques des patients montre qu'il n'existe pas de différence significative concernant l'âge moyen entre les patients ayant développé au moins un EIG par rapport à ceux qui n'en a pas développé. Ainsi, l'âge moyen est respectivement de 48.3 et 45.3 ans (p = 0.12) (Tableau 3). Cependant, le sexe masculin semble être un peu plus exposé au risque de développer un évènement indésirable lié aux soins (Tableau 3). Par ailleurs, notre étude montre que les EIG étaient plus fréquemment retrouvés chez les immunodéprimés et ceux ayant des antécédents d'hospitalisation durant les six derniers mois (p &#lt;10^-4^) (Tableau 3). L'exposition aux soins invasifs durant l'hospitalisation semble être prédictive de l'apparition d'évènements indésirables (Tableau 3).

**Tableau 3:** Facteurs de risque des évènements indésirables graves

	EIG présent (N = 162)	EIG absent (N = 1266)	*P*
Caractéristiques des patients			
Age moyen +/- DS (ans)	48,33 + /- 23,48	45,30 +/-23,78	0,126
Sexe masculin, n (%)	82(50,6)	536(42,3)	0.045
Antécédent d'hospitalisation dans les 6 derniers mois, n (%)	67 (41,4)	298 (23,5)	<10^-4^
Immunodépression, n (%)	69 (42,6)	324 (25,6)	<10^-4^
Caractéristiques de l'admission			
En urgence, n (%)	87(53,7)	494(39)	<10^-4^
Pendant la garde, n (%)	80(49,4)	399(31,5)	<10^-4^
Pendant le weekend ou jours fériés, n (%)	26(16)	134(10,6)	*0,038*
Exposition aux soins			
Exposition à au moins une procédure invasive	101 (62.3)	492 (38.9)	<10^-4^
Exposition à au moins un dispositif médical	151(93)	963(76,1)	<10^-4^
Exposition à au moins un produit de santé	161(99,4)	1 091(86,2)	<10^-4^

## Discussion

Nous avons procédé à une étude prospective d'incidence qui a duré un mois en 2009, réalisée auprès de tous les patients qui ont été hospitalisés dans 14 services du CHU Farhat Hached. Par ailleurs, nous avons prévu six retours de passage dans chaque service durant la période d'étude, selon un calendrier prédéfini en fonction de la disponibilité des référents désignés dans chaque service, afin de recueillir les différents soins administrés pour chaque patient et l'éventuel EIG apparu au cours de l'hospitalisation. Chaque évènement indésirable suspecté, par l'enquêteur en concertation avec le médecin traitant correspondant, a été confirmé ou infirmé secondairement lors de l'expertise médicale.

Par ailleurs, grâce au recueil prospectif, nous avons pu obtenir des informations complètes pour la quasi-totalité des patients, ce qui ne serait pas souvent le cas lors des études rétrospectives du fait d'un défaut de traçabilité dans les dossiers médicaux [17].

**Figure 2:**
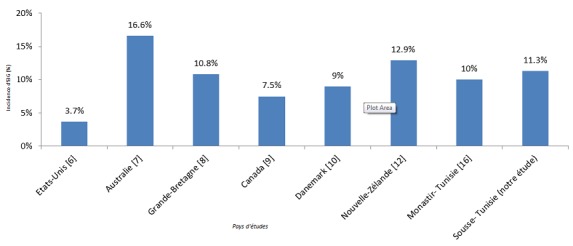
Comparaison de l'incidence d'évènements indésirables graves entre certains pays

Notre étude a permis d'estimer pour la première fois l'incidence d'au moins un EIG par patient au CHU F.Hached de Sousse chiffrée à 11,3% (IC 95% {9.6 - 12.9}). Cette incidence dépasse la médiane d'incidence des EIG rapportée dans la littérature qui vaut 9,2% [11]. Cependant, l'incidence en Australie et en Nouvelle Zélande était nettement supérieure par rapport à l'incidence retrouvée dans notre CHU (Figure 2).

Toutes les études considérées étaient, cependant, basées sur un recueil rétrospectif des données à partir du dossier médical [11], contrairement à notre étude, ainsi que l'étude française ENEIS [13] qui ont procédé à un recueil prospectif des données qui a prouvé ses avantages en terme d'identification des EIG, leur reproductibilité et leur fiabilité par rapport aux études rétrospectives ou transversales surtout si nous comptons étudier leur degré d'évitabilité et effectuer une analyse approfondie des causes racines [12, 18, 19]. Cependant, il reste à discuter un éventuel biais d'information (ou de mesure) en raison d'une réticence probable de l'équipe soignante et d'une méfiance dans le suivi médical des patients, étant donné que tous les services ont été informés de la période de l'enquête et de ses objectifs.

Notre étude confirme comme d'autres [13, 16] que ce sont les patients hospitalisés dans les unités de soins intensifs qui courent le plus haut risque de développer un EIG. Cependant, contrairement au CHU F. Bourguiba de Monastir (Tunisie) [16], l'incidence des EIG dans notre hôpital était plus élevée dans les services médicaux que chirurgicaux étant donné que le CHU F.Hached est un hôpital à vocation médicale plus que chirurgicale (3services chirurgicaux contre 11 services médicaux). Cette répartition s'explique également par la prédominance des services réputés à haut risque (service d'oncologie, d'hématologie, de cardiologie), en raison de la gravité clinique des patients hospitalisés dans ces services, le séjour relativement prolongé et la fréquence des procédures invasives à visée diagnostique et /ou thérapeutiques mais aussi le recours à des traitements agressifs tels que les immunosuppresseurs ou la corticothérapie au long cours. Etant donné que certains patients avec au moins un EIG étaient encore hospitalisés au moment de la fin du recueil des données, les conséquences observées dans notre étude ne sont que des estimations approchées de la réalité. Le taux de décès de 9,2% observé chez les patients ayant présenté au moins un EIG, bien que proche du taux de mortalité associé aux EIG apparus pendant l'hospitalisation en France (8,4%) [13], devrait aussi être interprété avec prudence vu que l'imputabilité de l'EIG dans la survenue de décès n'a pas été établie avec certitude. Les taux de décès associés aux EIG sont ainsi très différents entre les études variant de 4,9% en Australie [7, 17] à 27% aux Etats-Unis [20].

L'utilisation de la prolongation de séjour comme critère de gravité est commune à toutes les études réalisées sur le sujet [7, 10, 11, 13, 16, 21]. Ainsi, en Espagne, 3 200 journées d'hospitalisation supplémentaires (soit 6,1 journées supplémentaires par patient) ont été causées par la survenue d'un EIG dont 1 157 d'entre elles ont été jugées évitables [22]. Dans notre étude, 61,7% des EIG ont été à l'origine d'une prolongation de l'hospitalisation seule et 26% autres EIG ont mis en jeu le pronostic vital et par la suite ont prolongé indirectement l'hospitalisation. Cependant, l'utilisation de la prolongation de séjour comme critère de gravité est contestable pour trois raisons. En effet, ce critère de gravité est relativisé par l'état de santé du patient: par exemple la survenue d'une escarre de décubitus chez un patient en fin de vie ne prolongera pas la durée d'une hospitalisation alors que sa survenue chez un autre patient peut retarder une sortie programmée. En outre, ce critère dépend du moment de survenue de l'EIG: une infection urinaire nosocomiale diagnostiquée en début de séjour ne sera pas incluse alors qu'elle pourrait le prolonger d'un ou de deux jours si elle est diagnostiquée le jour de la sortie. La troisième raison est que ce critère n'a pas la même valeur pour les soignants et pour les gestionnaires pour lesquels il est plus directement associé à des enjeux économiques que cliniques [23].

La prédominance des infections nosocomiales dans notre hôpital avec une proportion de 45%, certes plus élevée que celle retrouvée dans les hôpitaux français et estimée à 24.1% [13, 23], doit attirer l'attention des décideurs à orienter leur politique de gestion des risques en renforçant leur programme de lutte contre les évènements indésirables de nature infectieuse.

En dehors des facteurs intrinsèques qui ont été largement étudiés dans la littérature et dont leur contribution à l'apparition d'EIG variait d'une étude à une autre [23–26], l'originalité de notre travail tient surtout dans l'identification de facteurs de risque extrinsèques, particulièrement reliés aux conditions d'admission et à l'exposition aux soins invasifs. En effet, les EIG étaient plus fréquemment observés chez les patients qui ont été hospitalisés pendant la garde, en urgence ou pendant les weekends et les jours fériés. Ce résultat est expliqué en partie, par la mauvaise organisation de travail liée à un manque en personnel soignant ajouté à un défaut de communication entre les professionnels de santé assumant la garde [16]. Le risque associé à l'admission pendant les week-ends ou les jours fériés n'était pas confirmé par certaines études [24, 27]. Récemment, certains auteurs se sont intéressés à d'autres aspects relatifs à l'admission, en particulier l'admission selon un mode urgent. Ainsi, notre résultat rejoint celui de l'étude menée par Bartlett et col. où le risque d'EIG augmenterait de 1,64*(p= 0,023)*en cas d'admission en urgence, expliqué, en partie, par la gravité de l'état clinique au moment de l'admission [25].

L'exposition aux procédures de soins invasifs (telle que la chirurgie, la biopsie, l'endoscopie…), aux dispositifs médicaux et aux produits de santé et le risque de développer un EIG a été bien documentée dans la littérature [11, 17, 23, 24, 28].

Malgré les limites de notre travail, on peut considérer que la réalisation d'une telle étude au CHU F.Hached laisse penser qu'il y a chez les responsables de l'hôpital une volonté de maîtrise de ces risques sanitaires. Cette dernière ne peut être réussie que grâce à une collaboration multidisciplinaire et à tous les niveaux avec la mise en place d'une gestion globale et coordonnée des risques qui exigera de l'ensemble des acteurs hospitaliers d'importants changements: responsabilisation de chaque acteur, modifications des pratiques, des comportements individuels et collectifs, et modifications des modes de décisions. Ces changements sont sous tendus par l′acquisition d′une culture commune de vigilance et de sécurité [29]. En effet, le développement d'une culture de sécurité des patients exige une formation et une éducation au préalable. Ainsi, il apparait, de plus en plus important, que la sécurité sanitaire soit enseignée très tôt dans le cursus des professions de santé pour que tous les soignants intègrent cette dimension durant leur vie. Dans ce cadre, l'OMS a élaboré un guide de formation des étudiants en médecine à la sécurité sanitaire permettant de les sensibiliser à ce concept dès le plus jeune âge [30].

## Conclusion

La sécurité du patient s'impose comme un sujet central des politiques de santé et devient l'affaire de tous à l'hôpital, quels que soient les domaines et niveaux de compétences des acteurs impliqués dans la chaîne des soins. Ainsi, afin d'améliorer la sécurité des patients, il est primordial de gérer les risques, ce qui consiste à prévenir leur réalisation ou s'ils se réalisent à réduire les conséquences dommageables et les pertes qui en résultent et enfin contrôler le coût des contentieux. Cependant, les risques visés par ce processus ne doivent pas être confondus avec les risques d'aléas thérapeutiques qui subsisteront toujours. La performance d'un établissement de santé est d'abord celle du niveau de sécurité garanti au patient. L'identification, la mesure et la prévention des risques évitables est un métier à part entière au service des patients qu'il s'agit de promouvoir et d'affirmer comme outil de pilotage et d'optimisation des compétences au travers de l'évaluation des activités de soins et des programmes de formation car ces EI sont fréquents, universels et leur incidence est très variable. Il serait intéressant de compléter ce travail par des études d'analyse des causes racines de ces EIG et des études d'évitabilité car des structures de gestion des risques, pourvues des moyens nécessaires, peuvent se révéler rapidement efficaces, évolutives et par suite indispensables. La mise en place d'un observatoire des EIG liés aux soins semble donc fondamentale pour atteindre ces objectifs.

## Annexes

**Annexe 1:** Critères de détection des évènements indésirables graves liés aux soins

**Annexe 2:** Exemples d'évènements indésirables graves
